# Vitamin D-Loaded Chitosan Nanostructures for Bone Regeneration: A Combined In Vitro and In Vivo Evaluation in an Osteoporotic Rat Model

**DOI:** 10.3390/medicina62010073

**Published:** 2025-12-29

**Authors:** Corina Giorgiana Muresan, Ioana Codruta Mirica, Alina Forray, Nausica Petrescu, Olga Soritau, Luciana-Mădălina Gherman, Simina Angela Lăcrimioara Iusan, Evelyn Vanea, Emilia Oprita, Ana Condor, Maria Aluas, Carmen Mihaela Mihu, Bianca Adina Boşca, Lavinia Patricia Mocan, Madalin Mihai Onofrei, Raluca Maria Pop, Bianca-Astrid Andone, Lucian Barbu-Tudoran, Sanda Boca, Mihaela Hedesiu, Patricia Ondine Lucaciu

**Affiliations:** 1 Department of Oral Health, Iuliu Hatieganu University of Medicine and Pharmacy, 400012 Cluj-Napoca, Romania; 2 Department of Community Medicine, “Iuliu Hațieganu” University of Medicine and Pharmacy, 400012 Cluj-Napoca, Romania; 3 Research Department, Prof. Dr. I. Chiricuta Oncology Institute, 400015 Cluj-Napoca, Romania; olgasoritau@yahoo.com; 4 Experimental Centre, Iuliu Hatieganu University of Medicine and Pharmacy, 400349 Cluj-Napoca, Romania; 5 Department of Morphofunctional Sciences (Histology), Iuliu Hațieganu University of Medicine and Pharmacy, 400012 Cluj-Napoca, Romania; 6 Department of Pharmacology, Toxicology and Clinical Pharmacology, Iuliu Hatieganu University of Medicine and Pharmacy, 400337 Cluj-Napoca, Romania; 7 Interdisciplinary Research Institute in Bio-Nano-Sciences, Babes-Bolyai University, 400271 Cluj-Napoca, Romaniasanda.boca@ubbcluj.ro (S.B.); 8 Department of Molecular Biology and Biotechnology, Faculty of Biology and Geology, Babes-Bolyai University, 400084 Cluj-Napoca, Romania; lucian.barbu@ubbcluj.ro; 9 National Institute for Research and Development of Isotopic and Molecular Technologies, 400293 Cluj-Napoca, Romania; 10 Department of Oral Radiology, Iuliu Hatieganu University of Medicine and Pharmacy, 400006 Cluj-Napoca, Romania

**Keywords:** bone regeneration, osteoporosis, chitosan, nanostructured scaffold, Vitamin D, tissue engineering, local drug delivery

## Abstract

*Background and Objectives*: Reduced bone quality due to osteoporosis significantly complicates oral rehabilitation and bone regeneration therapies. While Vitamin D (Vit. D3) is crucial for osteogenesis, systemic administration often lacks local efficacy. This study aimed to evaluate the osteoregenerative potential of a novel Chitosan-based nanostructured scaffold (NS) loaded with Vit. D3, underlining its efficacy in vitro and in an ovariectomized (OVX) rat model of osteoporosis. *Materials and Methods*: Chitosan NSs were fabricated with varying Vit. D3 concentrations. In vitro assessments included cytotoxicity (MTT assay), cell viability (Alamar Blue), and mineralization (Alizarin Red) using human dental follicle stem cells. In vivo, 30 Wistar rats were ovariectomized to induce osteoporosis (confirmed by biomarkers Osteocalcin and β-CTX) and were divided into three groups (n = 10). Bilateral maxillary bone defects were treated with (1) a Control (clot only), (2) a Hemostatic Sponge with Vit. D3 (HS/Vit. D3), or (3) an NS loaded with Vit. D3 (NS/Vit. D3-6.25 ng/mL). Histological and morphometric analyses were performed at 4 and 8 weeks. *Results*: In vitro, the NS loaded with 6.25 ng/mL Vit. D3 demonstrated superior cytocompatibility, achieving a cell viability of 117.77% at 72 h and significantly enhanced calcium nodule deposition compared to controls. In vivo, a total of 44 defect sites were analyzed following the exclusion of compromised samples (Control: 16 sites; HS/Vit. D3: 16 sites; NS/Vit. D3: 12 sites). The NS/Vit. D3-6.25 ng/mL group exhibited the highest degree of mature bone formation and vascularization (*p* < 0.05) compared to the Control and HS/Vit. D3 groups. While cellular activity (osteoblasts/osteocytes) was initially higher in the HS/Vit. D3 group, the NS/Vit. D3-6.25 ng/mL group achieved superior structural integration and scaffold replacement by mature bone tissue over time. *Conclusions*: The novel Vit. D3-loaded Chitosan NS effectively promotes bone regeneration in osteoporotic conditions. It supports osteogenic differentiation in vitro and enhances bone matrix maturation in vivo, suggesting its potential as a bioactive scaffold for regenerative dentistry.

## 1. Introduction

Alveolar bone loss in the maxilla and mandible, resulting from trauma, extractions, pathology, or congenital malformations, presents significant challenges for oral rehabilitation. Following tooth extraction, a rapid remodeling process occurs, often leading to a 40–60% reduction in alveolar ridge height and width within 2–3 years [1,2]. Given the increasing demand for dental implants, effective bone preservation and regeneration techniques have become fundamental to restorative dentistry [3].

Bone regeneration is a complex biological process requiring the migration and proliferation of specific osteoprogenitor cells to the defect site to synthesize new tissue [4]. However, successful regeneration depends not only on bone volume but also on bone quality. Defined by the National Institutes of Health (NIH) as “the sum of all characteristics of bone that influence the bone’s resistance to fracture” [5], bone quality encompasses architecture, turnover, mineralization, and micro-damage accumulation [6]. Compromised bone quality is frequently associated with systemic conditions such as osteoporosis and Vitamin D3 (Vit. D3) deficiency [7].

Osteoporosis is a global health issue characterized by reduced bone mass and micro-architectural deterioration, leading to fragility. While Dual-energy X-ray Absorptiometry (DXA) remains the standard for diagnosis, bone turnover biomarkers (BTMs) offer early assessment capabilities [4,8]. The complications of osteoporosis—compromised strength and accelerated bone loss—pose significant risks for regenerative procedures [9,10]. High-quality evidence supports the role of nutritional factors, specifically Vit. D3 and calcium, in mitigating these risks [10,11].

Vit. D3 plays a key regulatory role in bone regeneration through several concise mechanistic pathways. Acting as a steroid hormone, it maintains mineral homeostasis by enhancing intestinal calcium and phosphate absorption. At the bone level, Vit. D3 modulates remodeling by activating osteoclasts and osteoblasts and promoting the differentiation of mesenchymal stem cells into osteoblasts [10,12]. This suggests that maintaining adequate local levels of Vit. D3 is critical for the integration of bone grafts and subsequent mineralization.

Despite its positive effect on osteogenesis, the clinical utility of Vit. D3 in local regeneration is limited by its administration route. Systemic administration often fails to achieve sufficient local concentrations due to the short half-life of bioactive metabolites and potential systemic toxicity [13,14]. Consequently, there is a critical need for controlled local release systems capable of maintaining therapeutic concentrations of Vit. D3 directly at the defect site, particularly given that osteoblasts possess specific Vit. D3 receptors [12].

To address the limitations of systemic administration, recent studies have investigated various local delivery systems, including Vit. D3 conjugated gold nanoparticles [15], PLGA-based scaffolds [16], palmitic acid-based sterosomes [13], and calcitriol-loaded polylactic acid [17]. Among the available biomaterials, nanostructured scaffolds (NS) have emerged as a promising tool for regenerative therapy. Specifically, Chitosan (CS), a natural amino polysaccharide derived from chitin, demonstrates exceptional biocompatibility, biodegradability, and intrinsic antibacterial properties. Its molecular structure, similar to glycosaminoglycans found in the extracellular matrix of bone, makes it an ideal candidate for bioactive substance delivery [18,19]. The incorporation of therapeutic agents into the nano-scale roughness of CS scaffolds offers advantages such as high reproducibility, ease of use, and cost-effectiveness [20,21].

We hypothesize that a chitosan-based nanoscaffold delivery system loaded with Vit. D3 can stimulate the differentiation of stem cells into osteoblastic lineage, thereby enhancing osteoregeneration even within a compromised, osteoporotic microenvironment. Specifically, we theorize that, Vit. D3-loaded CS-NS will (1) support cytocompatibility and osteoblastic differentiation and mineralization in vitro and (2) improve bone regeneration outcomes in an ovariectomized (osteoporotic) rat model compared with conventional hemostatic sponges and untreated spontaneous healing.

## 2. Materials and Methods

### 2.1. Materials

Medium molecular weight Chitosan (CS), acetic acid (CH_3_COOH), Vit. D3 (Cholecalciferol), and polydimethylsiloxane (PDMS) were purchased from Sigma-Aldrich (St. Louis, MO, USA). Cell culture reagents, including 96-well and 24-well plates, were obtained from standard suppliers. For the in vivo study, Ketamine 10% solution (Kepro B.V., Deventer, The Netherlands) and Xylazine 2% solution (Bioveta, Ivanovice na Hané, Czech Republic) were used for anesthesia. Surgical supplies included resorbable sutures (LUXCRYL 910 6/0, Luxsutures, Luxembourg) and hemostatic gelatin sponge of 1 × 1 × 1 mm (Cutanplast, Dispotech, Gordona, Italy). Biochemical analysis was performed using Rat Osteocalcin and Rat β-CTX ELISA Kits (FineTest, Wuhan Fine Biotech Co., Ltd., Wuhan, China).

### 2.2. Preparation of CS-Based NS

CS NSs were fabricated via a solvent casting method. CS was dissolved in 10% acetic acid to achieve a final concentration of 20 mg/mL. A drop of the CS solution was cast onto a clean, nanopatterned PDMS mold surface and allowed to dry at 40 °C overnight. The PDMS mold was replicated from a silicon master fabricated via standard photolithography to ensure precise groove periodicity (415 nm) and depth (200 nm). After 24 h, the resulting nanostructured films (width = 5 mm, length = 5 mm, 0.13 mm thickness) were carefully detached and sterilized using β irradiation at 25 Gy for 15 min.

To obtain Vit. D3-loaded NS, a stock solution of Vit. D3 (100 µg/mL in phosphate-buffered saline) was diluted to a working concentration of 300 nM. Varying volumes of this solution (1.2, 2.5, 5, 7.5, and 10 µL) were dripped onto the sterilized NS to achieve specific final concentrations. Six experimental groups were established based on the Vit. D3 concentration, as detailed in Table 1.

### 2.3. In Vitro Characterization of the NS

#### Structural Analysis

The surface morphology and nanotopography of the fabricated NS were examined using Scanning Electron Microscopy (SEM). Prior to imaging, the samples were sputter-coated with a 7 nm layer of Platinum/Palladium (Pt/Pd) using an Agar Automated Sputter Coater (Agar Scientific, Stansted, UK) to enhance conductivity. High-resolution images were acquired using a Hitachi SU9320 Cold Field Emission Scanning Transmission Electron Microscope (CFEG-STEM) (Hitachi High-Tech, Tokyo, Japan), operating at an accelerating voltage of 30 kV. Images were captured at magnification levels ranging from 13.0 k to 25.0 k, as indicated in the figure legends.

### 2.4. Biological Evaluation

#### 2.4.1. Cell Culture

Adult MSCs derived from human dental follicle were used for all biological assays. The characterization of MSCs (positive for CD73, CD90, and CD105; negative for hematopoietic markers) was previously validated and is supported by established markers reported in earlier studies [22]. Cells were cultured in a standard medium (SM) consisting of DMEM (4.5 g glucose)/Ham’s F12 (1:1 ratio) supplemented with 15% Fetal Bovine Serum (FBS), two mM L-glutamine, 1% penicillin-streptomycin, 1% non-essential amino acids (NEA), 55 µM β-mercaptoethanol, and one mM sodium pyruvate, and maintained at 37 °C in a humidified atmosphere containing 5% CO_2_. All reagents were purchased from Sigma-Aldrich.

For osteogenic assays, an Osteogenic Medium (OS) was used, comprising DMEM High Glucose/F12 (phenol red-free) supplemented with 15% FBS, 1% NEA, 1% penicillin-streptomycin, two mM glutamine, 50 µg/mL ascorbic acid phosphate, 20 µM dexamethasone, and 10 mM β-glycerophosphate.

#### 2.4.2. Cytotoxicity Assay (MTT)

Cytotoxicity was evaluated according to ISO 10993-5 standards [22] using the MTT assay. The NS samples (0.1 g/mL) were incubated with the SM for 24 and 72 h at 37 °C. Cells were seeded in 24-well plates (7 × 10^4^ cells/well) and were treated with the conditioned medium, and controls were maintained in SM. At the end of the exposure period, the medium was removed, and 100 µL of MTT solution (1 mg/mL) was added to each well. Plates were incubated for 1 h at 37 °C in the dark to allow the formation of formazan crystals. The solution was then aspirated, and 150 µL of DMSO was added to dissolve the crystals. Absorbance was measured at 570 nm using a BioTek Synergy 2 microplate reader (BioTek Instruments, Winooski, VT, USA).

#### 2.4.3. Cell Viability (Alamar Blue)

Long-term cell viability was assessed using the Alamar Blue assay at 48 h. Cells were seeded in 24-well plates (7 × 10^4^ cells/well) on NS/Vit. D3-2.5 in either SM or OS, on NS/Vit. D3-0 and in SM, and compared with cells cultured on unloaded NS (NS/Vit. D3-0). After 48 h, the medium was replaced with 800 µL of fresh medium containing 10% Alamar Blue reagent. After 1 h of incubation, 200 µL aliquots were transferred to a 96-well plate, and fluorescence was measured at 560/590 nm (excitation/emission) using the BioTek Synergy 2 reader. All experiments were performed in triplicate, using distinct biological replicates to ensure reproducibility.

#### 2.4.4. Mineralization Assay (Alizarin Red Staining)

Calcium deposition was evaluated to assess osteogenic differentiation. Cells were seeded on the NS/Vit. D3-2.5 samples (5 × 10^4^ cells/well) in 24-well plates and cultured in OS for 14 days. The medium was refreshed every 3 days. On day 14, cells were fixed with 4% paraformaldehyde for 20 min, washed with PBS, and stained with 2% Alizarin Red S solution (pH 4.2). Qualitative assessment of mineralization was performed by visualizing and counting calcium nodules using a Zeiss Axiovert D1 inverted microscope equipped with an AxioCAM MRC camera (Carl Zeiss, Oberkochen, Germany).

Quantification of mineralization was performed using a 10% cetylpyridinium chloride (CPC) solution to solubilize calcium deposits. Briefly, mineralized matrices were incubated with CPC until complete dissolution of calcium-bound complexes was achieved. The resulting solutions were then analyzed by measuring the optical density at 562 nm using a BioTek Synergy™ 2 microplate reader. Tests were run in triplicate.

#### 2.4.5. Vitamin D3 Release Assay

The release of Vit. D3 was evaluated using cells cultured in the presence of NS/Vit. D3-2.5. The NS/Vit. D3 were loaded with a Vit. D3 concentration of 6.5 ng/mL and had a surface area of 25 mm^2^. Cells were cultivated under standard culture conditions, and the culture medium was collected to each medium change at predefined time points (72 h and 9 days).

Vit. D3 concentrations in the collected samples were quantified using a commercial enzyme-linked immunosorbent assay (ELISA) kit (Vitamin D3 ELISA Kit, Elabscience^®^, Elabscience Bionovation Inc., Houston, TX, USA), following the manufacturer’s instructions. Absorbance was measured at 450 nm using a microplate reader (Synergy™ 2, BioTek Instruments, Walpole, MA, USA).

A standard calibration curve was generated using known concentrations of Vit. D3 prepared in buffer solutions, and Vit. D3 release at each time point was calculated using the GraphPad 9 software.

### 2.5. In Vivo Experimental Design

#### 2.5.1. Ethical Statement and Animal Housing

The study protocol was approved by the Ethics Commission of Iuliu Hațieganu University of Medicine and Pharmacy Cluj-Napoca (Approval No. AVZ36/31.03.2023) and the Sanitary Veterinary and Food Safety Agency (Approval No. 361/28.04.2023). All procedures were conducted in strict accordance with the Directive 2010/63/EU on the protection of animals used for scientific purposes and followed the ARRIVE guidelines (Animal Research: Reporting of In Vivo Experiments).

Thirty female Wistar rats (8–12 weeks old; weight 200 ± 50 g) were obtained from the Center for Experimental Medicine and Practical Skills (UMF Biobase, Cluj-Napoca; Authorization No. 937/23.03.2022). Animals were housed in a controlled environment with a 12 h light/dark cycle and a temperature of 21 °C. Standard pellet food and water were provided ad libitum. The study was designed according to the 3Rs principles (Replacement, Reduction, and Refinement), ensuring the minimum number of animals required for statistical validity was used.

Given ethical and logistical constraints, we selected n = 10 animals per group (a total of 30 animals). We consider this sample size appropriate for an exploratory proof-of-concept study. The design, with two defect sites per animal, increases the number of measured sites and improves sensitivity. This approach aligns with the 3Rs principles (Replacement, Reduction, and Refinement), ensuring adequate statistical power to detect meaningful biological effects while minimizing animal use.

#### 2.5.2. Study Groups

**Figure 1:**
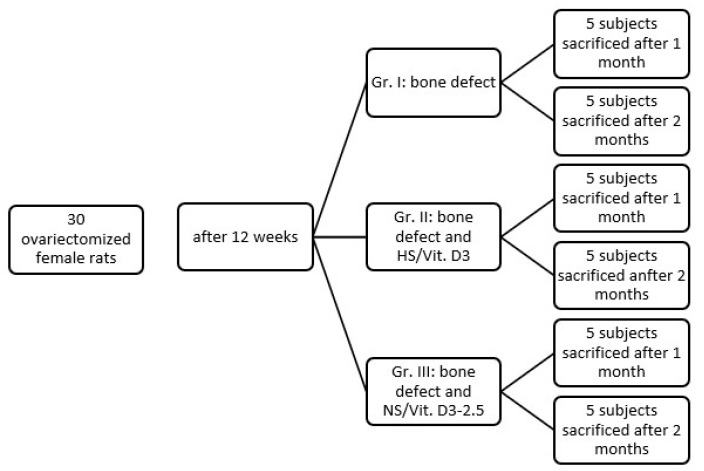
Distribution of study subjects (Wistar rats) in the study.

Following a one-week acclimatization period, the animals were randomly assigned to three experimental groups (n = 10 per group) using a simple randomization method based on unique ear tag identifiers (Figure 1):

1. Group I (Control): Ovariectomy (OVX) + Bone Defect + No Treatment (Clot only).
2. Group II (HS/Vit. D3): OVX + Bone Defect + Hemostatic Sponge soaked in 6.25 ng/mL Vit. D3 (the value of 6.25 ng/mL represents the equivalent of 2.5 µL).
3. Group III (NS/Vit. D3-2.5): OVX + Bone Defect + NS/Vit. D3-6.25 ng/mL.

#### 2.5.3. Surgical Protocol

Stage 1: Induction of Osteoporosis (Ovariectomy) Following health screening, all subjects underwent bilateral ovariectomy (OVX) to induce systemic osteoporosis. General anesthesia was induced via intraperitoneal injection of ketamine (0.1 mg/kg, Ketamine 10% solution; Kepro B.V., Woerden, Holland) in combination with xylazine (0.5 mg/kg, 2% solution; Xylazin Bio, Bioveta, Ivanovice na Hané, Czech Republic). The depth of anesthesia was carefully monitored throughout the procedure using multiple parameters, including the absence of the palpebral and pedal withdrawal reflexes, reduction in muscle tone, and evaluation of respiratory rate and depth. These indicators were assessed at standardized intervals to ensure adequate anesthesia and to prevent intraoperative pain perception or movement.

Animals were placed in a supine position, and their abdomen was shaved and disinfected. A ventral midline incision was performed to access the abdominal cavity. The ovaries were identified, ligated, and excised. Hemostasis was secured using electrocautery. The abdominal wall and skin were sutured in layers. Post-operative analgesia was provided using meloxicam (0.37 mg/kg) subcutaneously once daily for three days. Antibiotic prophylaxis with enrofloxacin (5%; 1 mg/animal/day) was administered for five days to prevent infection.

Stage 2: Alveolar Bone Defect Creation Twelve weeks post-OVX, after confirming osteoporotic status via bone turnover biomarkers, the animals underwent the second surgical phase. Under the same general anesthesia protocol, a full thickness mucoperiosteal flap was elevated on the mesial aspect of the maxillary first molar bilaterally.

**Figure 2:**
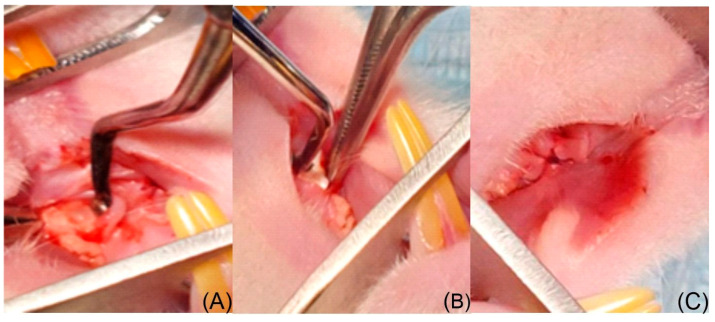
(**A**) Bone defect; (**B**) NS placement; (**C**) Defect closed by suturing the flap.

A standardized critical-sized bone defect (1 × 1 × 1 mm) was created using a sterile round bur under continuous saline irrigation to prevent thermal necrosis. Both bilateral defects in each animal were treated with the same assigned experimental condition (Control, HS/Vit. D3, or NS/Vit. D3-2.5) to avoid intra-subject confounding effects. Surgeries were performed using 2.5× magnification loupes. The defects were treated according to the assigned group (Control, HS/Vit. D3, or NS/Vit. D3-2.5). The flaps were repositioned and closed with resorbable 6/0 sutures (Luxsutures). Post-operatively, analgesics were administered via drinking water, and animals were monitored daily for signs of infection or dehiscence (Figure 2). 

#### 2.5.4. Humane Endpoints and Euthanasia

Animals were monitored continuously. Any subject displaying signs of severe distress (e.g., hypothermia, bradycardia) that could not be managed was euthanized immediately as a humane endpoint. At the designated time points (4- and 8 weeks post-defect surgery), animals were euthanized by anesthetic overdose (Ketamine 150–200 mg/kg and Xylazine 10–15 mg/kg).

### 2.6. Histological and Morphometric Analysis

#### 2.6.1. Sample Processing

Tissue samples were harvested at 4 weeks (1 month) and 8 weeks (2 months) post-surgery. Symmetrical blocks (2 × 2 × 2 cm) of the left and right maxillae were excised and fixed in 10% neutral buffered formalin for 48 h. Decalcification was performed using Osteodec (Bio-Optica, Milan, Italy), a buffered disodium EDTA solution, for 72 h to facilitate calcium chelation without compromising cellular integrity.

Following macroscopic identification of the defect site, samples were processed for paraffin embedding. To optimize visualization, the right maxillae were sectioned longitudinally along the axis of the defect, while the left maxillae were sectioned perpendicularly. Serial sections of 4 µm thickness were cut and stained with Hematoxylin and Eosin (H&E) for light microscopy. For each defect, three non-consecutive sections were selected and analyzed, corresponding to three sections per sample.

#### 2.6.2. Microscopic Assessment

Slides were examined using a Leica DM750 microscope equipped with an ICC50 digital camera (Leica Biosystems, Nußloch, Germany) and analyzed using LAS EZ software (Version 3.4, Leica Biosystems). All histological and morphometric evaluations were performed by a general pathologist strictly blinded to the experimental group assignments. Note that blinding was not feasible during the surgical procedure due to the visible differences in the graft materials.

#### 2.6.3. Morphometry and Histological Scoring

Quantitative morphometric analysis was performed to measure the residual defect size. The maximum length and width of the defect were measured microscopically, and the defect surface area was calculated (Area = Length × Width).

Qualitative histological assessment was conducted using a semi-quantitative scoring system adapted from Solchaga et al. [23] and Lucaciu et al. [24]. The scoring criteria evaluated 17 distinct parameters across three domains:

1. Bone Architecture: Presence of osteoporosis, bone bridging, trabecular thickness, and the ratio of immature to mature bone.
2. Cellular Response: Presence and localization of osteoblasts, osteocytes, osteoclasts, and vascularization.
3. Graft Interaction: Inflammation, presence of granulation tissue, graft degradation, and replacement by new bone.

Each parameter was scored on a scale (e.g., 0–2 or 0–3) based on the extent and localization of the tissue response (peripheral vs. central). The detailed scoring system is provided in Table 2.

### 2.7. Statistical Analysis

Data were recorded in Microsoft Excel and analyzed using the Social Science Statistics software package [25]. Normality of data distribution was assessed using the Shapiro–Wilk test.

For the in vitro assays the statistical analysis was performed with the *t*-test, continuous variables with normal distribution, such as animal body mass and morphometric defect measurements, are presented as mean +/− standard deviation (SD). Inter-group comparisons for these variables were performed using the independent samples *t*-test or One-way Analysis of Variance (ANOVA). Intra-group comparisons (weight changes over time) were analyzed using a paired-samples *t*-test.

For the morphometric analysis of residual defect size, the ANOVA included 16 defect sites in Group I, 16 in Group II, and 12 in Group III. Normality of the data distribution was confirmed using the Shapiro–Wilk test prior to parametric analysis.

The Mann–Whitney U test was employed to assess differences in histological scores between groups, as these data were ordinal and non-normally distributed. Statistical significance was set at a *p*-value of < 0.05.

## 3. Results

### 3.1. Structural Characterization of the NS

**Figure 3:**
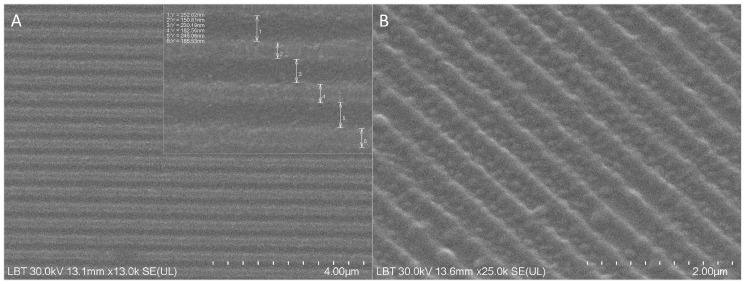
Scanning Electron Microscopy (SEM) analysis of the NS. (**A**,**B**) Micrographs at increasing magnification illustrating the uniform nanopatterning.

The surface morphology of the fabricated NS was analyzed by SEM to verify the fidelity of the nanopatterning process. The micrographs revealed a highly ordered surface topography characterized by equally spaced parallel rectangular grooves. Image analysis confirmed a structural periodicity of 415 ± 11 nm. The nanogrooves presented a mean width of 242 ± 8 nm, a wall thickness of 173 ± 14 nm, and a depth of 200 nm. This specific nanotopography was designed to mimic the natural architecture of the bone extracellular matrix, providing physical cues for osteoblast alignment (Figure 3).

### 3.2. In Vitro Biological Assessment

#### 3.2.1. Cell Viability

**Figure 4:**
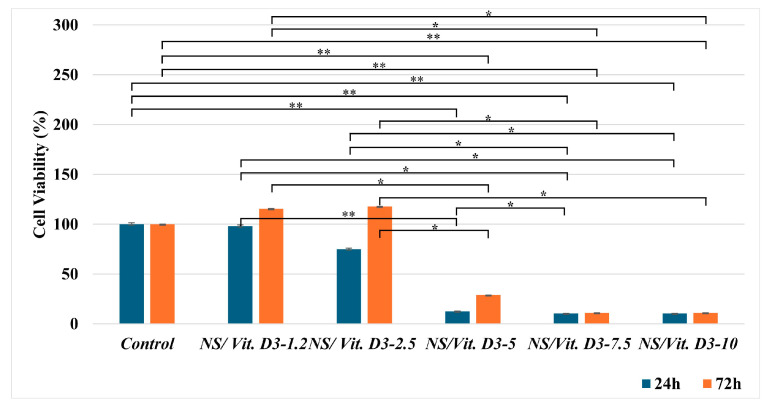
Cell viability of mesenchymal stem cells assessed by MTT assay at 24 and 72 h (* *p* < 0.05, ** *p* < 0.01).

The MTT assay revealed a time- and dose-dependent response to the Vit. D3-loaded NS. At 24 h, a decrease in cell viability for the NS loaded with Vit. D3 12.5 ng/mL, with 87.5%, for the one loaded with 18.75 ng/mL and with 25 ng/mL with 89. 69% occurred. The NS containing 3 ng/mL showed a cell viability of 97.91% and the one with 6.25 ng/mL a growth of 75%. At 72 h, groups with higher Vit. D3 concentrations (NS/Vit. D3-5.0, -7.5, and -10) exhibited severe cytotoxicity, with viability dropping below 30%. Conversely, the NS/Vit. D3-2.5 group showed a significant increase in cell activity, reaching 117.77% viability compared to the control (Figure 4). This elevation above 100% suggests a potent proliferative effect induced by the synergistic action of the scaffold and Vit. D3. Based on these results, the NS/Vit. D3-2.5 formulation was selected for all subsequent differentiation and in vivo experiments.

#### 3.2.2. Cell Proliferation (Alamar Blue)

**Figure 5:**
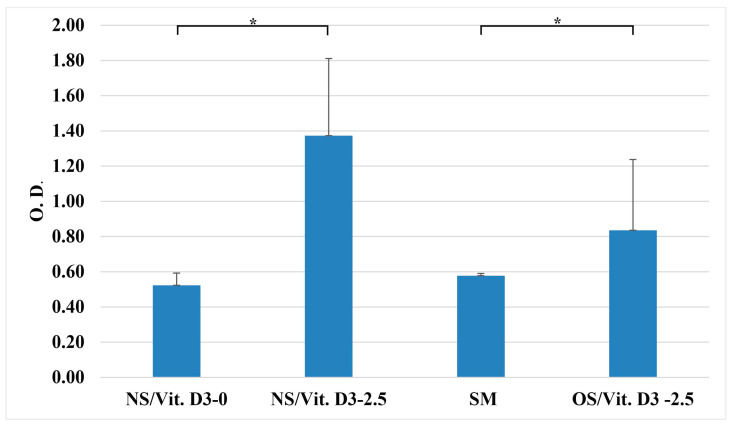
Alamar Blue cell viability assessment (* *p* < 0.05).

The Alamar Blue assay further confirmed the proliferative potential of the selected NS. Cells cultured on NS/Vit. D3-2.5 in OS exhibited a significantly higher reduction rate of resazurin to resorufin compared to the unloaded control (NS/Vit. D3-0) at 48 h (*p* < 0.05). This indicates enhanced metabolic activity and cell proliferation induced by local Vit. D3 release (Figure 5).

#### 3.2.3. Osteogenic Differentiation (Alizarin Red Staining)

**Figure 6:**
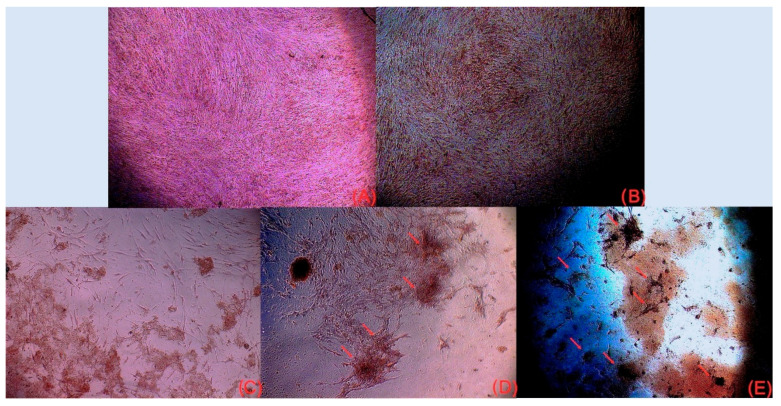
Alizarin Red S staining of calcium deposits after 14 days of culture. (**A**) Cells in OS (Control); (**B**) Cells in OS supplemented with soluble Vit. D3 (6.25 ng/mL); (**C**) Cells in SM seeded on unloaded NS (NS/Vit. D3-0); (**D**) Cells in OS seeded on unloaded NS (NS/Vit. D3-0) (the arrows indicate the mineralization nodules.); (**E**) Cells in OS seeded on NS/Vit. D3-2.5 (the arrows indicate the mineralization nodules).

Qualitative assessment of mineralization at 14 days revealed that the NS itself supports osteogenesis, as evidenced by the formation of four distinct mineralization nodules in the unloaded group (Figure 6C). However, the addition of Vit. D3 significantly enhanced this effect. The NS/Vit. D3-2.5 group displayed seven distinct mineralization nodules (Figure 6E).

**Figure 7:**
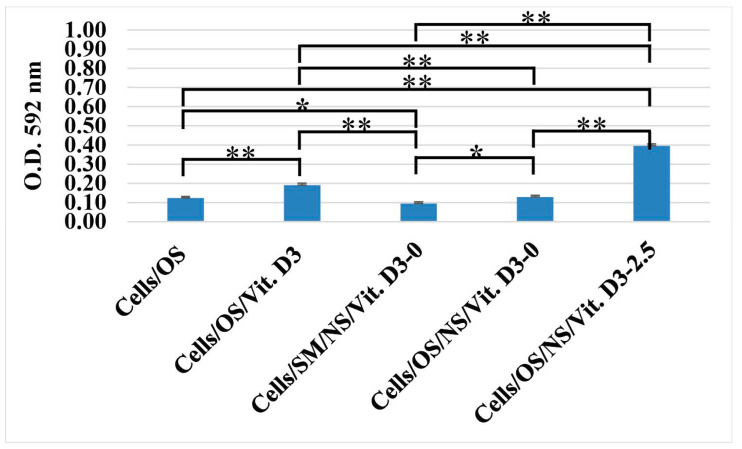
Quantification of Alizarin Red S staining (* *p* < 0.05, ** *p* < 0.01).

The quantification of the alizarin red staining assay reinforces the observation that the NS by itself induces mineralization, but the adding of Vit. D3 enhance this property. As shown in Figure 7, NS/Vit. D3 records the best results, showing statistical difference to all other groups.

#### 3.2.4. Vitamin D3 Release

**Figure 8:**
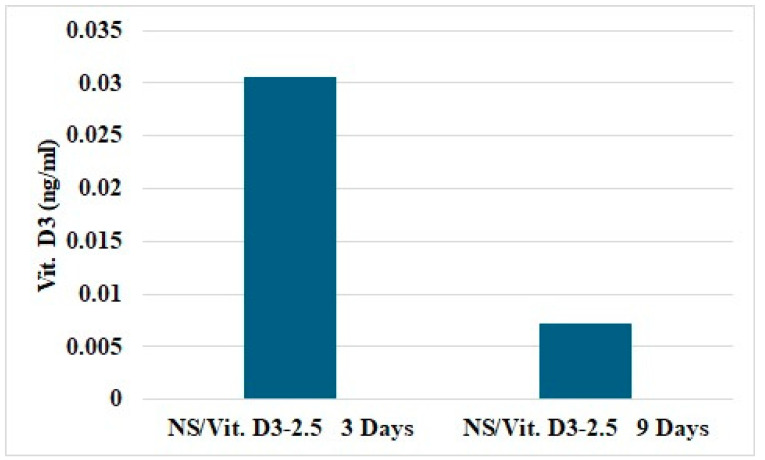
The release of Vit. D3 from NS/Vit. D3-2.5.

Drug release analysis revealed a time-dependent release of Vit. D_3_ from NS/Vit. D3-2.5. After 3 days of culture, a higher concentration of Vit. D_3_ was detected in the collected medium, reaching 0.03 ng/mL indicating an initial release phase. At a later time point (9 days), a decrease in the detection of Vit. D_3_ release concentration was observed, meeting only 0.07 ng/mL with lower levels measured after 9 days of culture. These results indicate that NS/Vit. D3-2.5 enables sustained Vit. D_3_ release under standard cell culture conditions, with a more pronounced release occurring in the first 3 days. at earlier time points (Figure 8).

### 3.3. In Vivo Evaluation

#### 3.3.1. Assessment of Bone Turnover Biomarkers

The systemic osteoporotic status was further validated by quantifying plasma bone turnover markers before ovariectomy (T0) and 12 weeks post-ovariectomy (T1).

**Figure 9:**
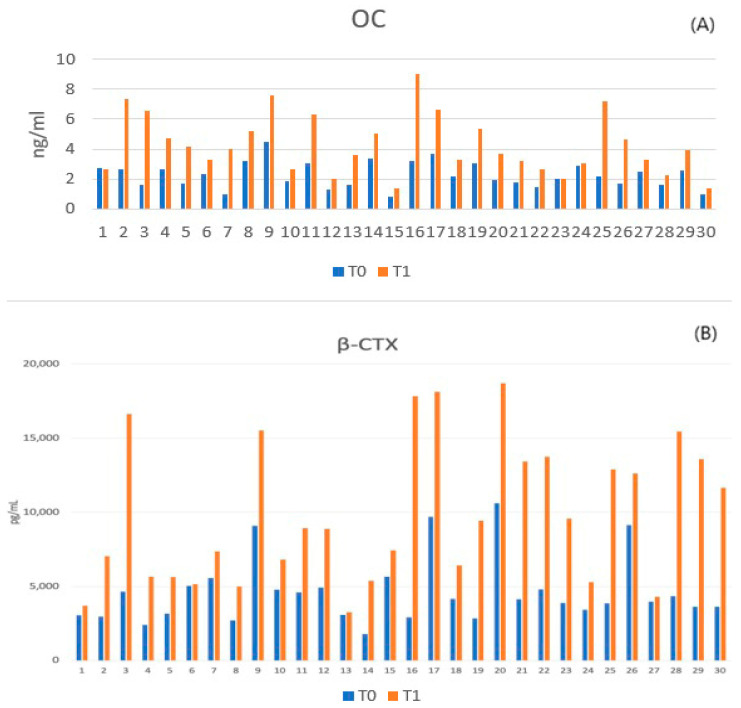
Graphical representation of systemic bone turnover biomarkers. (**A**) Osteocalcin levels and (**B**) β-CTX levels significantly increased 12 weeks post-ovariectomy, confirming a state of high-turnover osteoporosis prior to the creation of the bone defects.

As presented in Table 3, a marked increase in both bone formation (Osteocalcin) and bone resorption (β-CTX) markers was observed at T1 compared to baseline (T0). The level of β-CTX, a specific marker of bone resorption, more than doubled over the 12-week period. This simultaneous elevation of formation and resorption markers is characteristic of the high-turnover osteoporosis induced by estrogen deficiency, confirming the suitability of the animals for the defect regeneration model (Figure 9).

#### 3.3.2. Clinical Observations and Model Validation

Two animals from the Control group were lost due to anesthesia-related complications during the initial surgery. Following ovariectomy, all surviving subjects demonstrated a rapid and statistically significant increase in body weight (t = 16.22, *p* < 0.001), confirming the systemic metabolic changes associated with estrogen deficiency and validating the induction of the post-menopausal model.

#### 3.3.3. Macroscopic Evaluation and Defect Morphometry

A total of 44 bone defect sites were available for analysis. Initial group sizes were n = 10 animals (20 sites) per group. Two subjects in Group I died during the study (loss of 4 sites). Subsequently, specific samples from Group II (4 sites) and Group III (8 sites) were excluded due to improper fixation or tissue necrosis. The final distribution was: Group I (16 sites), Group II (16 sites), and Group III (12 sites).

Macroscopic examination of the harvested maxillae revealed varying degrees of defect closure. Morphometric analysis of the residual defect surface area indicated a progressive reduction in defect size from the Control group to the NS/Vit. D3-2.5 group. The mean residual defect areas were:

- Group I (Control): 0.34 ± 0.19 mm^2^;
- Group II (HS/Vit. D3): 0.28 ± 0.13 mm^2^;
- Group III (NS/Vit. D3-2.5): 0.21 ± 0.10 mm^2^.

Although the NS/Vit. D3-2.5 group exhibited the smallest residual defect area, suggesting a trend toward accelerated osseous bridging, the differences between groups did not reach statistical significance (*p* = 0.115, One-way ANOVA).

#### 3.3.4. Histopathological Analysis

##### Local Bone Architecture and Mineralization

**Figure 10:**
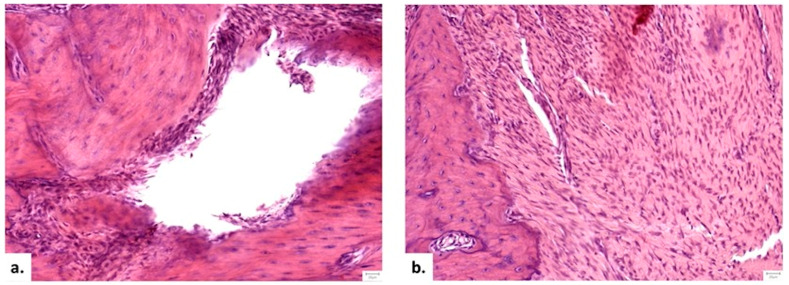

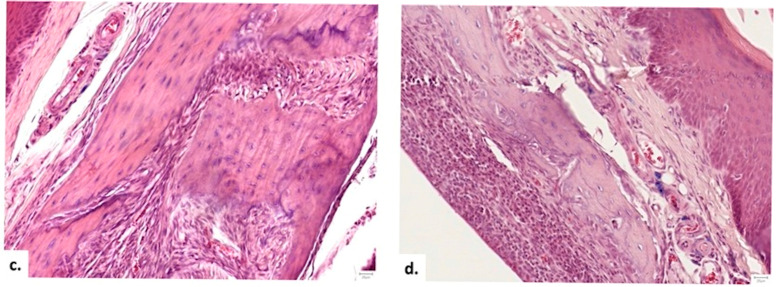
Histological progression of defect healing (H&E stain, 20×). (**a**) Group I (Control, 1 month): Absence of bone, with the defect occupied by fibrous connective tissue and a central void. (**b**) Group II (HS/Vit. D3, 1 month): Dense fibrous tissue rich in fibroblasts. (**c**) Group III (NS/Vit. D3-2.5, 1 month): association of dense irregular connective tissue and osseous tissue; (**d**) Group III (NS/Vit. D3-2.5, 2 months): Advanced healing showing organized osseous tissue filling the defect.

Histological examination confirmed the osteoporotic status of the animals, with Group I (Control) displaying thin, disconnected trabeculae indicative of severe local bone compromise. In contrast, the therapeutic groups (II and III) exhibited only mild osteoporotic features within the defect area (Figure 10). The final scores obtained following the histopathological assessment are presented in Table 4.

Analysis of bone matrix maturation revealed significant differences. Group III (NS/Vit. D3-2.5) demonstrated the highest frequency of mature bone formation, significantly superior to both the Control and HS/Vit. D3 groups (*p* = 0.016, Mann–Whitney U). While bone formation at the graft surface was significantly higher in both treated groups compared to control (*p* = 0.023), the NS/Vit. D3-2.5 promoted a more advanced state of remodeling.

##### Defect Bridging and Healing Patterns

**Figure 11:**
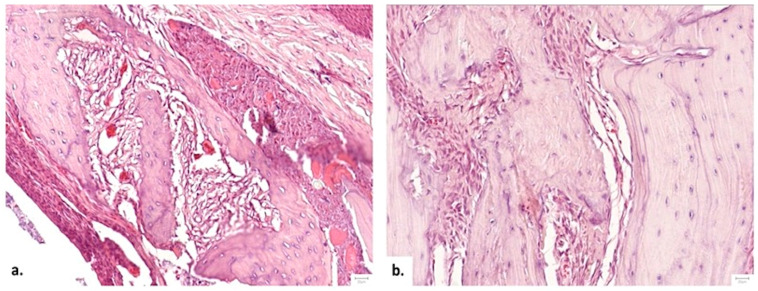
Comparison of bridging patterns. (**a**) Thin, delicate bone bridge with limited trabeculae at 1 month. (**b**) Thick bone bridge with robust trabeculae lined by osteoblasts at 2 months in the NS/Vit. D3-2.5 group.

Bone bridging was absent in 83.3% of Control samples. Conversely, all subjects in the therapeutic groups (II and III) exhibited bridging (*p* = 0.001 vs. Control). Notably, while both groups formed bridges, Group III (NS/Vit. D3-2.5) produced thick bone bridges in 66.7% of cases, compared to only 33.3% in Group II (HS/Vit. D3), suggesting superior structural integrity (Figure 11).

##### Cellular Response and Vascularization

**Figure 12:**
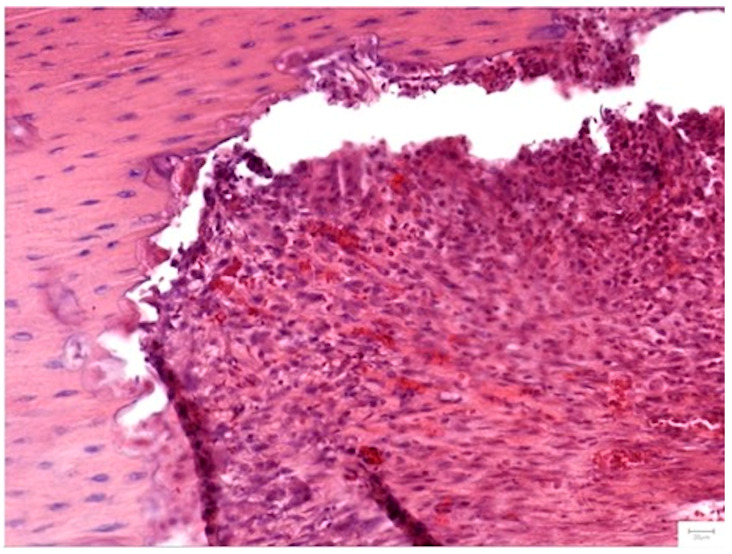
Vascularization in Group III (NS/Vit. D2-2.5) at 1 month (H&E, 20×). The defect shows organized and centrally penetrating capillaries, consistent with the enhanced angiogenic response. Dense connective tissue surrounds the newly formed vessels.

The cellular dynamics varied by treatment. Group II (HS/Vit. D3) showed significantly higher scores for osteoblasts (*p* = 0.045 at 1 month) and osteocytes (*p* = 0.025) compared to Group III, indicative of an intense early metabolic burst. However, Group III (NS/Vit. D3-2.5) exhibited superior vascularization at the 1-month mark (*p* = 0.045), with capillaries penetrating the center of the graft in all samples (Figure 12). This early angiogenesis likely supported the subsequent maturation of the bone matrix observed at 2 months.

##### Graft Integration and Safety

**Figure 13:**
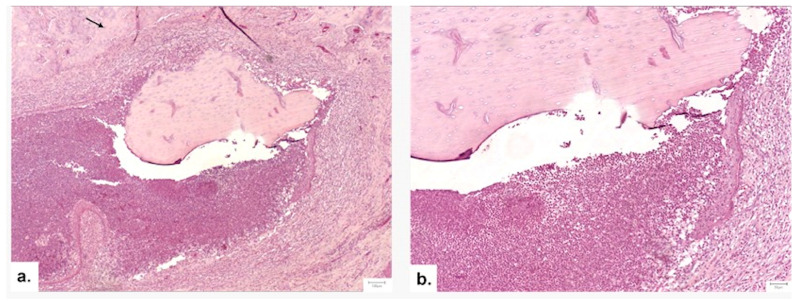
Histological evidence of complication (Group III, 1 month). (**a**,**b**) A bone sequestrum surrounded by a dense inflammatory infiltrate of polymorphonuclear neutrophils, indicative of localized osteomyelitis (H&E, 5× and 10×).

Inflammation was significantly lower in the therapeutic groups compared to the Control (*p* = 0.033 at 1 month). Complete degradation of the scaffold was observed in all subjects by the endpoint. Notably, the replacement of graft material with mature bone was most extensive in Group III (*p* = 0.042), confirming the biodegradability and osteoconductivity of the NS. Complications were rare; two isolated cases of abscess/sequestrum formation were noted (one in Group II, one in Group III) and excluded from scoring (Figure 13).

## 4. Discussion

### 4.1. In Vitro Performance

Vit. D3 is an essential micronutrient for maintaining bone mineral density, offering significant potential for the prevention and treatment of osteoporosis [26,27]. Numerous studies have confirmed its pivotal role in osseointegration, suggesting that Vit. D3 deficiency impairs bone regeneration and may compromise implant stability [28].

In clinical practice, Vit. D3 is predominantly administered orally to treat systemic deficiency. Literature suggests that oral supplementation prior to dental implant insertion may promote osseointegration by increasing general bone density [29]. However, the efficacy of this approach relies heavily on the patient’s metabolic and hormonal status. As an alternative, local application strategies have been explored, such as implant surface modifications to enhance bone-to-implant contact [30], or single topical applications. Nevertheless, the latter approach is often considered insufficient to predictably stimulate bone regeneration due to rapid washout [31].

Tissue engineering literature establishes that scaffolds should ideally mimic the structure of native tissue to fulfill their biological function [32]. Our study utilized NS with indentations of 200 nm depth and rectangular grooves with a periodicity of 415 ± 11 nm. These dimensions classify the material as a porous scaffold with macro-pores [33]. This specific nanotopography was selected to mimic the natural architecture of the bone extracellular matrix, which provides critical physical cues for osteoblast alignment and differentiation [34,35].

CS a glucosamine polymer derived from the deacetylation of chitin, was selected for its demonstrated osteogenic properties, as well as its biocompatibility, biodegradability, and antibacterial activity [36,37,38]. Furthermore, CS has the potential to stimulate osteoblast adhesion and proliferation, providing the mechanical properties necessary to support the regeneration process [39].

The MTT assay confirmed the suitability of our specific NS formulation. The NS/Vit. D3-2.5 showed no cytotoxicity at 24 h, adhering to the ISO 10993-5 standard [22]. Notably, at 72 h, this group exhibited a 17.77% increase in cell viability compared to the control. These results are comparable to, or exceed, those of other established biomaterials for bone regeneration [40,41,42]. Since other concentrations proved to be toxic according to the ISO standard [22], all subsequent assays focused on the NS/Vit. D3-2.5 formulation.

Alizarin Red staining quantification revealed that the NS significantly promotes extracellular matrix mineralization, a defining feature of late-stage osteogenic differentiation. The increased calcium deposition observed in NS-based cultures suggests that the NS provides a bioactive microenvironment conducive to mineral nucleation and growth, consistent with previous reports demonstrating the role of biomaterial surfaces in directing osteogenic mineralization [43,44]. Furthermore, the pronounced mineralization observed when the NS was combined with Vit. D3 indicates a synergistic effect, whereby biochemical stimulation and material-derived cues cooperatively enhance osteogenic maturation [45].

The synergy between Vit. D3 and CS was further highlighted by the Alamar Blue assay. Cell proliferation on the NS/Vit. D3-2.5 scaffold was significantly higher than on the unloaded NS or in standard osteogenic medium supplemented with Vit. D3. This aligns with literature stating that Vit. D3 enhances cell proliferation [46], while the presence of CS itself stimulates cell division [47]. Finally, the differentiation potential was evidenced by calcium staining; the presence of Vit. D3 on the NS increased the number of mineralization nodules from 4 to 7, a phenomenon supported by similar findings in recent studies [48,49].

The Vit. D3 release study demonstrated that NS/Vit. D3-2.5 provides a time-dependent release profile under standard cell culture conditions. The higher concentration of Vit. D3 detected after 3 days indicates a desorption of surface-associated molecules or diffusion-driven release from nanostructured systems [50,51]. This early availability of Vit. D3 is particularly relevant for osteogenic applications, as Vit. D3 is known to play a crucial role during the early stages of osteoblast differentiation and matrix maturation [52].

The reduced Vit. D3 concentration detected at later time points suggests a controlled and prolonged release behavior rather than a rapid burst release. Such release kinetics are advantageous for bone tissue engineering strategies, as sustained exposure to osteoinductive factors has been shown to promote osteogenic differentiation while minimizing potential cytotoxic effects associated with high local concentrations [53,54]. The observed release profile indicates that NS/Vit. D3-2.5 can maintain bioactive levels of Vit. D3 over an extended period, supporting long-term osteogenic stimulation.

Furthermore, the controlled release of Vit. D3 from NS/Vit. D3-2.5 may contribute to the enhanced mineralization observed in scaffold-based cultures, as Vit. D3 regulates calcium homeostasis and osteoblast-specific gene expression, including osteocalcin and alkaline phosphatase [55]. The combination of material-derived cues and sustained biochemical stimulation provided by the NS supports the concept that NS/Vit. D3-2.5 acts as an active delivery platform rather than a passive carrier.

Overall, these findings highlight the potential of NS/Vit. D3-2.5 as an effective system for controlled Vit. D3 delivery in bone-related applications, aligning with current strategies that emphasize the integration of bioactive molecule release with advanced biomaterial design to enhance osteogenic outcomes [56,57].

### 4.2. In Vivo Performance in the Osteoporotic Model

We utilized an animal model with induced osteoporosis, a condition known to significantly reduce bone regeneration capacity. While the specific effects of systemic osteoporosis on jawbone density remain a subject of debate, growing evidence from both preclinical and clinical studies indicates a strong correlation between general bone mineral density (BMD) and maxillary bone quality. Retrospective research suggests that osteoporosis may adversely affect the outcomes of pre-prosthetic bone grafting or sinus augmentation procedures [58].

To simulate this challenging biological context, we implemented a 12-week post-ovariectomy latency period. Literature confirms that this timeframe is sufficient for the onset of characteristic osteoporotic bone changes [59,60,61,62,63,64]. This framework allowed us to test our intervention in a realistic and compromised regenerative environment. Our histological analysis and bone turnover biomarker evaluation confirmed the presence of osteoporosis, justifying the timing of the defect induction. However, it must be noted that animal models have inherent limitations when extrapolating results to human clinical contexts.

### 4.3. Comparative Efficacy and Mechanisms

Macroscopic examination revealed varying degrees of defect closure. Morphometric analysis indicated a numerical trend toward reduced residual defect surface area in the NS/Vit. D3-2.5 group compared to controls. However, it is crucial to note that these differences did not reach statistical significance (*p* = 0.115). We attribute this discrepancy to the distinct physicochemical properties of the carriers. Gelatin sponges are known for their rapid degradation and high porosity, which may facilitate an immediate but disorganized cellular influx. In contrast, the nanostructured scaffold likely dictates a more controlled, slower infiltration, favoring the structural maturation of the tissue over an initial burst of cellularity [65]. Consequently, while these morphometric data are consistent with the histological evidence of enhanced bone matrix quality, they cannot independently establish superior efficacy regarding the rate of defect closure.

Overall, the NS/Vit. D3 provided improved efficiency compared to the HS/Vit. D3. We attribute this effect to both the nanoscale surface topography and the material itself; CS is known for its hygroscopic properties [66], which likely enhance fluid absorption and biological interaction at the defect site.

### 4.4. Clinical Implications and Future Directions

Compared with well-established methods, such as autografts and allografts, which remain the gold standard but carry risks of infection, morbidity, and poor integration [30,63], the use of functional biomaterials, such as Chitosan-based NS, offers a viable alternative. Our results complement the research directions proposed by García-Gareta et al. (2015) [67] and Wang et al. (2020) [68], who emphasize the need for “smart” biomaterials with controlled delivery. The novelty of our study lies in integrating Vitamin D into a bioactive matrix that not only supports regeneration but accelerates it even under the adverse conditions of osteoporosis.

### 4.5. Limitations and Methodological Considerations

The interpretation of these results requires consideration of several methodological constraints inherent to the study design. Primarily, while the bilateral defect model effectively doubled the number of analytical sites, the final sample size was impacted by histological processing artifacts. Specifically, the exclusion of compromised samples from Groups II and III due to improper fixation reduced the statistical power of the morphometric analysis. Although the remaining data were sufficient to delineate clear histological trends, a larger cohort would be required to achieve statistical significance across all morphometric parameters.

Furthermore, this study focused on the biological endpoint of regeneration rather than the physicochemical kinetics of the delivery system.

Ethical considerations regarding animal welfare influenced the experimental architecture. In strict adherence to the 3Rs principle, particularly Reduction, we prioritized the comparison of the novel system against the standard of care (hemostatic sponge) and spontaneous healing. We acknowledge that the exclusion of an unloaded scaffold control and a gold-standard barrier membrane limits our ability to fully decouple the effects of the chitosan matrix from the Vit. D3 payload. Instead, validation of the osteoporotic model relied on comparisons between baseline (T0) and post-ovariectomy (T1) biomarkers and rigorous alignment with established latency periods reported in the literature [59,60,61,62,63,64]. While this approach is ethically sound and scientifically valid, the inclusion of a sham control would have provided a more granular baseline for assessing the intervention’s systemic effects. Future confirmatory studies will incorporate these additional controls to benchmark efficacy against established clinical modalities.

From an analytical perspective, the histopathological assessment was predominantly semi-quantitative. The utilization of standard light microscopy limited our ability to perform high-precision digital morphometry or volumetric quantification. To overcome this in future protocols, we intend to implement automated digital image analysis and utilize specific differential stains, such as Masson’s Trichrome, to provide a more rigorous quantitative distinction between mineralized matrix, osteoid, and fibrous tissue.

Finally, the study design prioritized the evaluation of the novel nanostructure against a standard hemostatic carrier. It did not, however, include a positive control group utilizing a clinical gold-standard barrier membrane (e.g., a collagen membrane such as Bio-Oss/Bio-Gide) over the Vitamin D-soaked sponge. Including such a comparison would have allowed for a more precise decoupling of the scaffold’s barrier function from its bioactive properties. Future studies are warranted to benchmark the efficiency of this nanostructured system against these established clinical modalities over extended observation periods.

## 5. Conclusions

The present study demonstrates that an NS loaded with 6.25 ng/mL Vit. D3 exerts a significant osteoregenerative effect in an osteoporotic rat model, superior to both spontaneous healing and a standard hemostatic carrier. Our findings suggest that the synergistic interaction between the scaffold’s nanotopographical cues and the local delivery of Vit. D3 promotes not only cellular recruitment but also the structural maturation and vascularization of the bone matrix. Consequently, this bioactive system represents a promising therapeutic strategy for regenerative dentistry, particularly for patients with compromised bone quality. Although these findings are encouraging, they remain preliminary and limited to an animal model. Therefore, any translational relevance should be interpreted with caution. Further studies, including mechanistic investigations, larger preclinical cohorts, and rigorously designed clinical trials, are necessary to determine whether similar benefits could be achieved in human clinical settings.

## Figures and Tables

**Table 1:** Experimental concentrations of Vit. D3-loaded NS.

Group Code	Vitamin D3 Volume (μL)	Final Concentration (ng/mL)
NS/Vit. D3-0	0	0
NS/Vit. D3-1.2	1.2	3
NS/Vit. D3-2.5	2.5	6.25
NS/Vit. D3-5.0	5	12.5
NS/Vit. D3-7.5	7.5	18.75
NS/Vit. D3-10	10	25

Note: The final concentration in ng/mL was calculated based on the volume of the 300 nM stock solution applied to the standard scaffold surface. Values represent the final concentration of the loading solution applied to the scaffold surface, not the released concentration.

**Table 2:** Histopathological assessment and scoring of samples, with emphasis on the bone defect area and alterations induced by the synthetic graft.

No.	Parameter	Score	Microscopic Aspect
1.	Osteoporosis	0	Not discernable
		1	Subtle
		2	Evident
2.	Bone formation at the surface of the graft	0	Absent
		1	Present at the periphery
		2	Present in the center
		3	Present at the periphery and in the center
3.	Bone formation in the graft area	0	Absent
		1	Present in the surface of the graft area
		2	Present in the center of the graft area
4.	Bone bridge	0	Absent
		1	Thin
		2	Thick
5.	Bone trabeculae	0	Absent
		1	Present at the periphery
		2	Present in the center
		3	Present at the periphery and in the center
6.	Immature bone	0	Present at the periphery and in the center
		1	Present in the center
		2	Present at the periphery
		3	Absent
7.	Mature bone	0	Absent
		1	Present at the periphery
		2	Present in the center
		3	Present at the periphery and in the center
8.	Osteoblasts	0	Absent
		1	Present at the periphery
		2	Present in the center
		3	Present at the periphery and in the center
9.	Osteocytes	0	Absent
		1	Present at the periphery
		2	Present in the center
		3	Present at the periphery and in the center
10.	Osteoclasts	0	Absent
		1	Present at the periphery
		2	Present in the center
		3	Present at the periphery and in the center
11.	Havers canals	0	Absent
		1	Present at the periphery
		2	Present in the center
		3	Present at the periphery and in the center
12.	Inflammation	0	Present, abundant
		1	Present, scant
		2	Absent
13.	Vascularization	0	Absent
		1	Present in the surface of the graft area
		2	Present in the center of the graft area
14.	Granulation tissue	0	Present
		1	Absent
15.	Graft	0	Detected
		1	Not detected
		na	Not applicable
16.	Osteoclastic degradation of the graft	0	Absent
		1	Present at the periphery
		2	Present in the center
		3	Present at the periphery and in the center
		na	Not applicable
17.	Replacement of graft by mature bone	0	Absent
		1	Present at the periphery
		2	Present in the center
		3	Present at the periphery and in the center
		na	Not applicable

**Table 3:** Comparison of serum bone turnover biomarkers before (T0) and 12 weeks after (T1) ovariectomy.

Bone Biomarker	T0 (Baseline)	T1 (12 Weeks Post-OVX)	Trend
Osteocalcin (ng/mL)	2.26 ± 0.85	4.28 ± 1.96	Increased Turnover
β-CTX (pg/mL)	4609.10 ± 2214.88	9842.55 ± 4766.71	Increased Resorption

**Table 4:** Frequency distribution of histological healing scores across study groups at 1 month (1) and 2 months (2) post-surgery. Data represents the frequency (0–1) of samples exhibiting specific features.

Histological Parameter	Microscopic Aspect	Gr I-1	Gr I-2	Gr II-1	Gr II-2	Gr III-1	Gr III-2
Osteoporosis	Not discernable	0	0	0.67	0.67	0.67	0.67
	Subtle	0.5	1	0.33	0.33	0.33	0.33
	Evident	0.5	0	0	0	0	0
Bone formation (Surface)	Absent	0	0.17	0.1	0	0	0
	Peripheral	0.6	0.83	0.4	0.33	0.33	0.34
	Central	0	0	0	0	0	0
	Central & Peripheral	0.4	0	0.5	0.67	0.67	0.67
Bone formation (Depth)	Absent	0	0	0	0	0	0
	Surface	0	0	0.3	0.33	0.33	0.17
	Profound	0	0	0.7	0.67	0.67	0.83
Bone Bridge	Absent	0.8	1	0	0.17	0.17	0.17
	Thin	0.2	0	0.6	0.33	0.33	0.16
	Thick	0	0	0.4	0.5	0.5	0.67
Bone Trabeculae	Absent	0	0.17	0	0	0	0
	Peripheral	0.8	0.83	0	0.17	0.17	0.33
	Central	0	0	0	0	0	0
	Central & Peripheral	0.2	0	1	0.83	0.84	0.67
Immature Bone	Absent	0.2	0	0.4	0.5	0.5	0.33
	Peripheral	0	0	0	0	0	0
	Central	0.8	1	0.5	0.5	0.5	0.5
	Central & Peripheral	0	0	0.1	0	0	0.17
Mature Bone	Absent	0.3	0.33	0.1	0	0	0
	Peripheral	0.4	0.67	0.7	0.5	0.5	0.33
	Central	0.1	0	0	0	0	0
	Central & Peripheral	0.2	0	0.2	0.5	0.5	0.67
Osteoblasts	Absent	0	0	0	0	0	0
	Peripheral	0.6	1	1	0.2	0.5	0.33
	Central	0	0	0	0	0	0
	Central & Peripheral	0.4	0	0	0.8	0.5	0.67
Osteocytes	Absent	0	0	0	0	0	0
	Peripheral	0.6	1	0	0.17	0.17	0.33
	Central	0	0	0.1	0	0	0
	Central & Peripheral	0.4	0	0.9	0.83	0.83	0.67
Osteoclasts	Absent	0.6	0.83	0.9	1	1	0.5
	Peripheral	0.4	0.17	0.1	0	0	0.5
	Central	0	0	0	0	0	0
	Central & Peripheral	0	0	0	0	0	0
Haversian Canals	Absent	0.7	1	0	0.33	0.33	0
	Peripheral	0.2	0	0.1	0.33	0.33	0.33
	Central	0	0	0	0	0	0
	Central & Peripheral	0.1	0	0.9	0.33	0.33	0.67
Inflammation	Present (Abundant)	0.1	0.17	0	0.17	0.17	0
	Present (Scant)	0.5	0.33	0.1	0.33	0.33	0.17
	Absent	0.4	0.5	0.9	0.5	0.5	0.83
Vascularization	Absent	0	0	0	0	0	0
	Surface of graft	0.4	0.67	0.5	0	0	0
	Depth of graft	0.6	0.33	0.5	1	1	1
Granulation Tissue	Absent	0	0.5	0.1	0.33	0.33	0
	Present	1	0.5	0.9	0.67	0.67	1
Graft Remnant	Detected	n/a	n/a	0	0	0	0
	Not detected	n/a	n/a	1	1	1	1
Scaffold Degradation	Absent	n/a	n/a	0	0	0	0
	Peripheral	n/a	n/a	0	0	0.5	0.33
	Central & Peripheral	n/a	n/a	1	1	0.5	0.67
Scaffold Replacement	Absent	n/a	n/a	0	0	0	0
	Peripheral	n/a	n/a	0.7	1	0.5	0.33
	Central & Peripheral	n/a	n/a	0.3	0	0.5	0.67

Note: Data values represent the relative frequency (0.0 to 1.0) of samples exhibiting a specific feature within each group (e.g., a value of 0.67 indicates the feature was present in 67% of the samples). Column headers denote Group and Time point (e.g., Gr I-1 = Group I at 1 month; Gr I-2 = Group I at 2 months).

## Data Availability

The datasets generated and analyzed during the current study are available from the corresponding author upon reasonable request.

## Abbreviations

The following abbreviations are used in this manuscript:

β-CTX	Beta-Crosslaps (C-terminal telopeptide of type I collagen)
ANOVA	Analysis of Variance
ARRIVE	Animal Research: Reporting of In Vivo Experiments
BMD	Bone Mineral Density
BTMs	Bone Turnover Biomarkers
CFEG-STEM	Cold Field Emission Scanning Transmission Electron Microscope
CS	Chitosan
DMEM	Dulbecco’s Modified Eagle Medium
DMSO	Dimethyl Sulfoxide
DXA	Dual-Energy X-ray Absorptiometry
ECM	Extracellular Matrix
EDTA	Ethylenediaminetetraacetic Acid
FBS	Fetal Bovine Serum
H&E	Hematoxylin and Eosin
HS	Hemostatic Sponge
ISO	International Organization for Standardization
MSCs	Mesenchymal Stem Cells
MTT	3-(4,5-dimethylthiazol-2-yl)-2,5-diphenyltetrazolium bromide
NEA	Non-Essential Amino Acids
NS	Nanostructured Scaffold
OC	Osteocalcin
OS	Osteogenic Medium
OVX	Ovariectomy
PBS	Phosphate-Buffered Saline
PDMS	Polydimethylsiloxane
PLGA	Polylactic-co-glycolic Acid
Pt/Pd	Platinum/Palladium
SD	Standard Deviation
SEM	Scanning Electron Microscopy
SM	Standard Medium
TCP	Tissue Culture Plastic
UV	Ultraviolet
Vit. D/Vit. D3	Vitamin D/Vitamin D3 (Cholecalciferol)
